# Supplementary material for: GSE1 links the HDAC1/CoREST co-repressor complex to DNA damage
Source: Nucleic Acids Res. 2023 Oct 25;51(21):11748–69. doi: 10.1093/nar/gkad911 (PMC10681733; doi:10.1093/nar/gkad911)
Supplement: gkad911_Supplemental_Files [file gkad911_supplemental_files.zip › Supplementary Material.pdf]

Supplementary Information

GSE1 links the HDAC1/CoREST co-repressor complex to DNA damage

Terezia Vcelkova, Wolfgang Reiter, Martha Zylka, David M. Hollenstein, Stefan Schuckert, Markus Hartl and Christian Seiser

Supplementary Figures S1-S6

Supplementary Tables S1-S5

Supplementary Figure S1

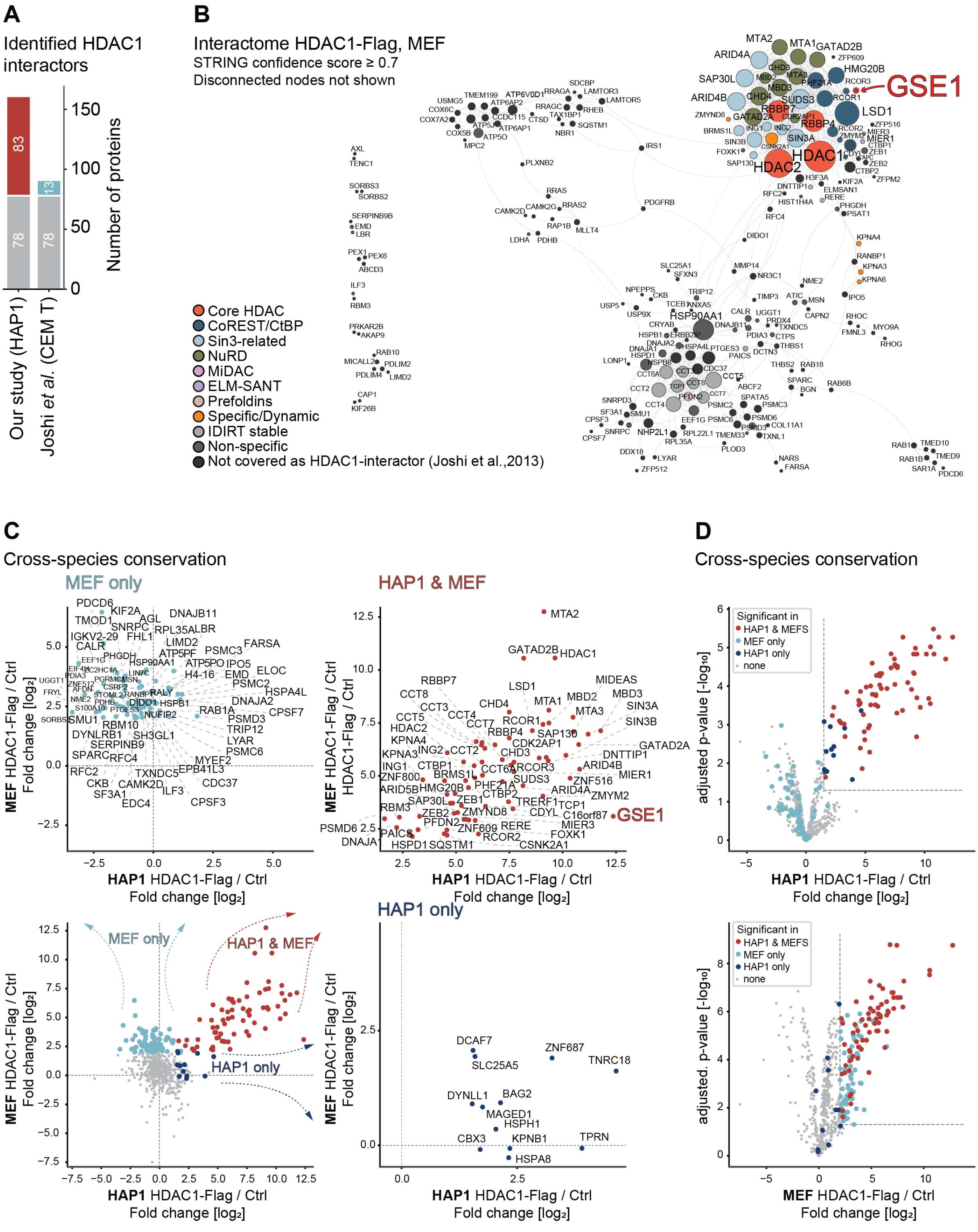

Supplementary Figure S1

(A) Bar plot displaying number of proteins interacting with HDAC1 identified by Joshi *et al.* (47) and our study. The red and blue boxes represent interactors found only in our study or by Joshi *et al.* **(B)** STRING DB-based protein interaction network of the HDAC1 interactome in mouse embryonic fibroblasts (MEF). High-confidence interactions (confidence score ≥ 0.7) were selected. Disconnected nodes are not shown. The interactors were classified into functional protein groups in accordance with Joshi *et al.* (47). Strength of the associations are represented by the thickness of the edges. **(C)** Scatter plots illustrating cross-species conservation of the HDAC1 interactome determined by AP-MS in MEF and HAP1 cells. Proteins designated as interactors (as described in panel D) only in MEF cells are shown in light blue, only in HAP1 cells are shown in dark blue, and in both cell types are shown in red. **(D)** Volcano plot displaying the interactome of FLAG-tagged HDAC1 in HAP1 cells (top) and MEF cells (bottom) determined by AP-MS. Proteins designated as interactors only in MEF cells (≥ 2 log2-fold change [FC], adjusted p-value ≤ 0.05) are shown in light blue, only in HAP1 cells (≥ 1.5 log2-fold change [FC], adjusted p-value ≤ 0.05) are shown in dark blue, and in both cell types are shown in red.

Supplementary Figure S2

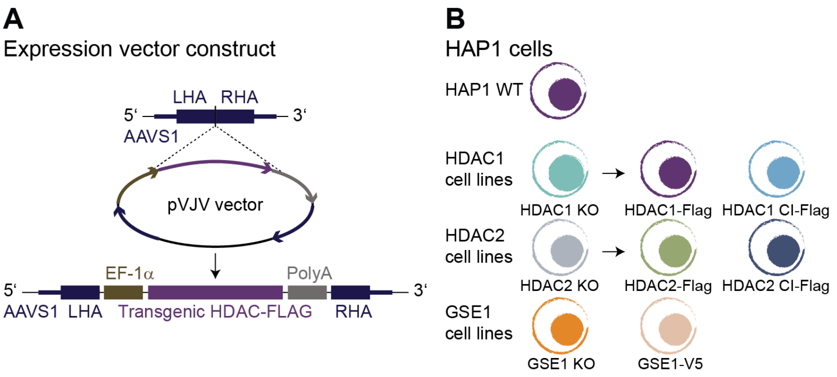

Supplementary Figure S2

(A) Illustration of targeting strategy for establishment of transgenic cell lines expressing wildtype or inactive flag-tagged versions of HDAC1 or HDAC2. The transgenes (purple) were cloned into pVJV vector and integrated into safe harbour AAVS1 locus within HAP1 genome (dark blue) by CRISPR/Cas9 technology (LHA – left homology arm, RHA – right homology arm). The expression of transgenes was under the control of EF-1a promoter (army green). **(B)** Schematic illustration of cell lines used in this study. Original HAP1 WT cell line was used to generate HDAC1, HDAC2 and GSE1 knockout cell lines as well as the V5-tagged GSE1 cell line. Transgenic cell lines expressing wildtype and inactive versions of HDAC1 or HDAC2 were generated in their respective knockout backgrounds.

Supplementary Figure S3

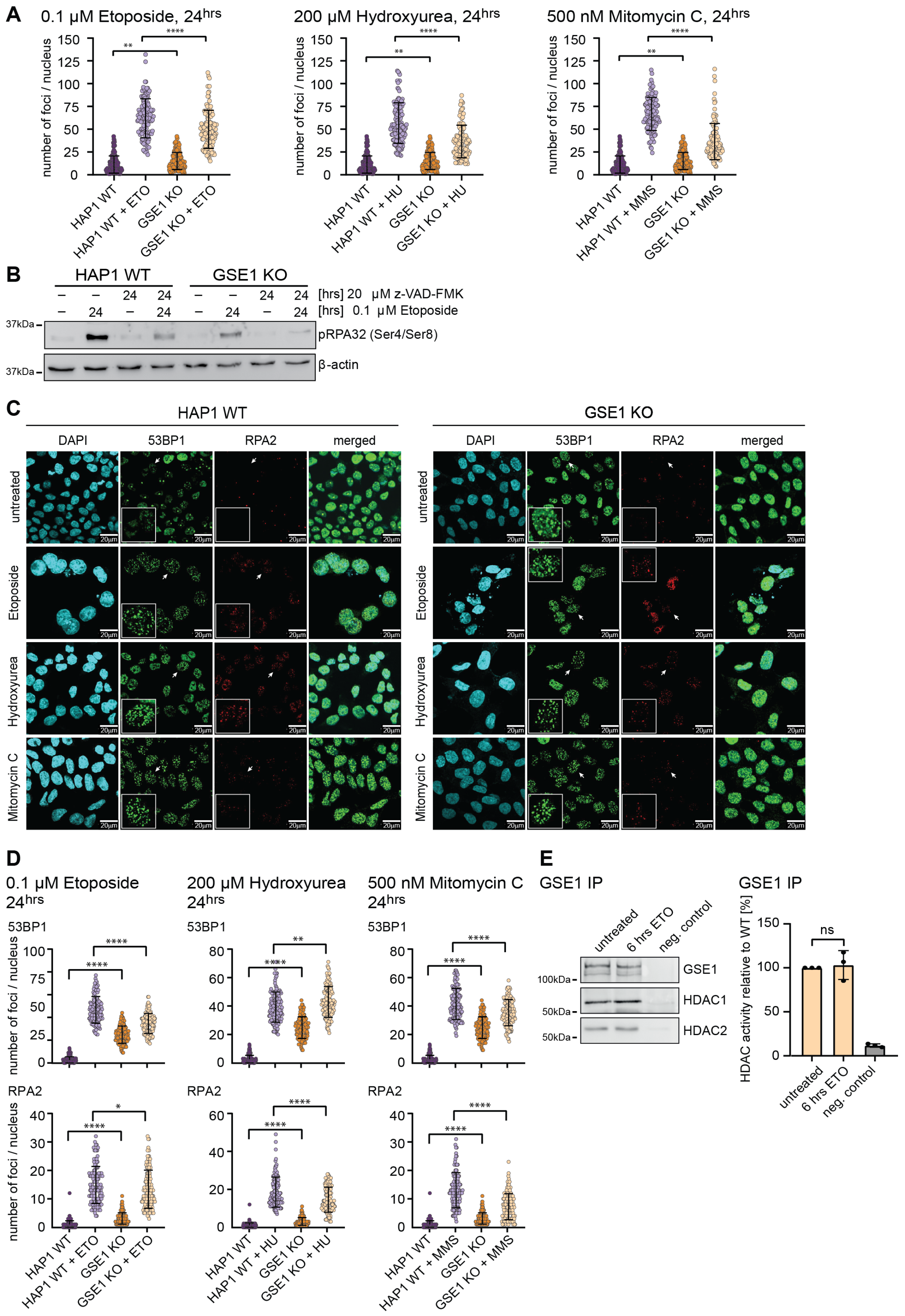

Supplementary Figure S3

(A) Dot plot illustrating γH2AX foci quantification upon etoposide, mitomycin C or hydroxyurea treatment. 40 nuclei were counted per genotype and condition. The data represent all counted foci ± standard deviation (SD) of 3 biological replicates. Significance was determined by unpaired parametric t-test by comparison to wildtype. *$p < 0.05$, **$p < 0.01$, ***$p < 0.001$. **(B)** Western blot illustrating RPA Ser4/Ser8 phosphorylation in HAP1 WT and GSE1 KO cells, either treated or not with the caspase inhibitor z-VAD-FMK and/or etoposide for the indicated durations. Antibodies used for detection are depicted on the right. **(C)** Formation of RPA or 53BP1 foci was analysed by immunofluorescence staining for RPA (red) and 53BP1 (green). The nucleus was stained with DAPI. The arrow indicates the selected enlarged cell. **(D)** Dot plot illustrating RPA or 53BP1 foci quantification upon etoposide, mitomycin C or hydroxyurea treatment. 40 nuclei were counted per genotype and condition. The data represent all counted foci ± standard deviation (SD) of 3 biological replicates. Significance was determined by unpaired parametric t-test by comparison to wildtype. *$p < 0.05$, **$p < 0.01$, ***$p < 0.001$. **(E)** Western blot illustrating immunoprecipitated endogenous GSE1 complexes in HAP1 WT cells treated with etoposide for 6 hours or left untreated. Antibodies used for detection are depicted on the right. Negative control: pull-downs performed using anti-mouse IgG antibodies. Effect of etoposide treatment on deacetylase activity of GSE1-HDAC complexes in HAP1 WT cells is shown in the bar graph. The data represent the mean values ± standard deviation (SD) of 3 biological replicates. Deacetylation activity obtained with untreated HAP1 WT extracts was set to 100%. Significance was determined by unpaired parametric t-test by comparison to wildtype. *$p < 0.05$, **$p < 0.01$, ***$p < 0.001$.

Supplementary Figure S4

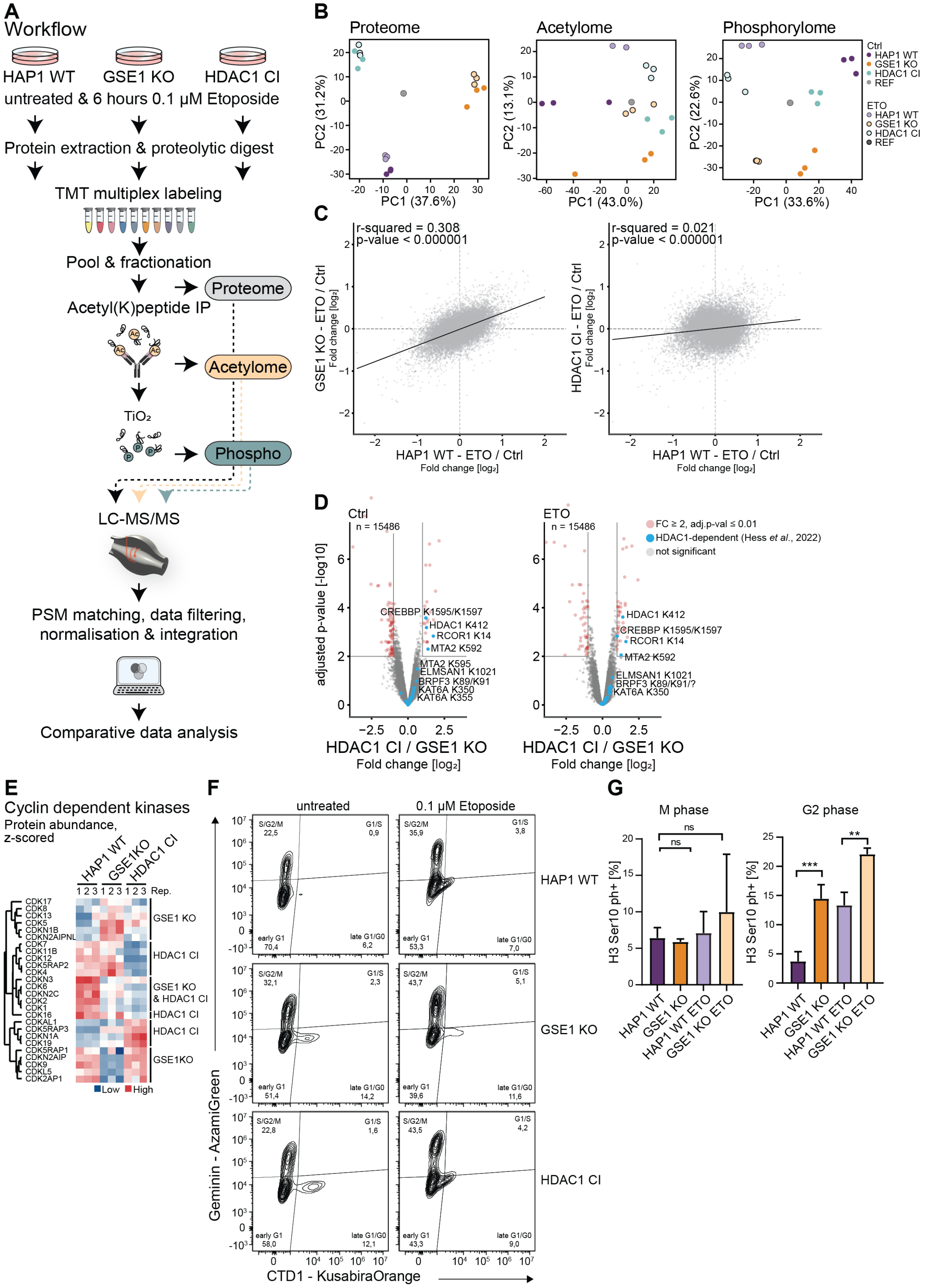

Supplementary Figure S4

(A) Quantitative mass spectrometry workflow. Cells were treated for 6 hours with etoposide or left untreated, proteins were extracted and digested by trypsin. Peptides were labelled TMTpro 10plex, pooled and fractionated using high pH HPLC. Proteome aliquots were removed for MS measurement. Acetylated peptides were enriched by acetyl-lysine immunoprecipitation. Acetyl-IP flowthrough was used for enrichment of phosphorylated peptides by titanium dioxide precipitation. Finally, proteomes, acetylomes and phosphorylomes were analysed by LC-MS/MS. After data processing, the phosphorylation and acetylation sites were normalised to protein abundance and the effects of HDAC1 inactivation and GSE1 ablation were assessed in comparative analysis of untreated and etoposide treated conditions. **(B)** Principal Component Analysis (PCA) plots illustrating relatedness of proteome, acetylome and phosphorylome datasets. Acetylome profiles of untreated HAP1 WT and GSE1 KO samples showed higher variance between replicas. **(C)** Scatter plot comparing etoposide affected acetylation between HAP1 wildtype cells and GSE1 KO cells (right) or HDAC1 CI cells (left), related to Figure 4B. **(D)** Volcano plots displaying acetylome differences between HDAC1 CI and GSE1 KO cells, either in untreated conditions (left) or upon 6 hours treatment with 0.1 µM etoposide (right). Significantly different acetylation sites are shown in red (≥ 2-fold change, adjusted p-value ≤ 0.01), HDAC1-dependent acetylation sites previously described by Hess *et al.* (36) are shown in blue. **(E)** Heatmap showing the protein abundance profiles of selected cyclin dependent kinases in untreated HAP1 WT, GSE1 KO, and HDAC1 CI cells. Corresponding gene names are indicated on the left. Values are z-scored. **(F)** Representative flow cytometry plots showing the cell cycle distribution of HAP1 WT, HDAC1 CI and GSE1 KO cells untreated or treated with etoposide for 24 hours. The data represent the percentage of cells in each cell cycle phase ± standard deviation (SD) of 3 biological replicates. Significance is determined by unpaired parametric t-test by comparison to wildtype. *p < 0.05, **p < 0.01, ***p < 0.001. **(G)** Immunofluorescence staining of H3 Ser10 phosphorylation quantified by counting of positive cells either in M-phase (robust signal in nucleus) and G2 phase (foci in the nucleus) are depicted. The data represent the percentage of cells in each M and G2 phase ± standard deviation (SD) of 3 biological replicates. On average 250 nuclei were counted per focal view. Significance is determined by unpaired parametric t-test by comparison to wildtype. *p < 0.05, **p < 0.01, ***p < 0.001.

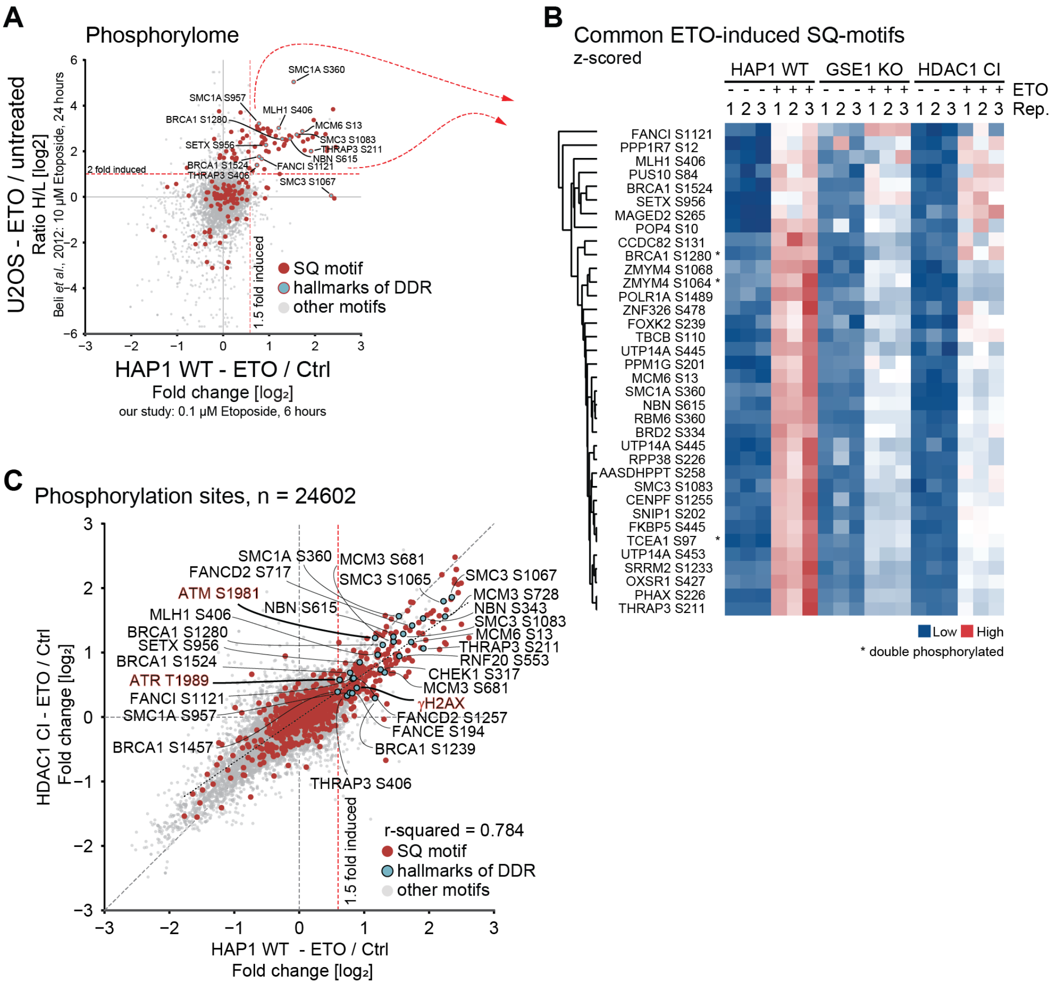

Supplementary Figure S5

(A) Scatter plot of phosphorylation sites commonly quantified in our study and the study of Beli *et al.* (48), comparing the effects of long time (24 hours) and short time (6 hours) etoposide treatment. Sites harbouring an SQ motive are shown in red, sites representing hallmarks of the DDR are shown in blue. **(B)** Heatmap illustrating phosphorylation profiles in HAP1 WT, GSE1 KO and HDAC1 CI cells of SQ sites induced by 6 hours and 24 hours etoposide treatment (dataset of Beli *et al.* (48), cut-off ≥ 2-fold change, our study ≥ 1.5-fold change). * marks quantification of phosphorylation sites arising from double phosphorylated peptides where only a single site was confidently assigned. **(C)** Scatter plot comparing etoposide affected phosphorylation between HAP1 wildtype cells and HDAC1 CI cells, related to Figure 5A. Sites harbouring an SQ motive are shown in red, sites representing hallmarks of the DDR are shown in blue.

Supplementary Figure S6

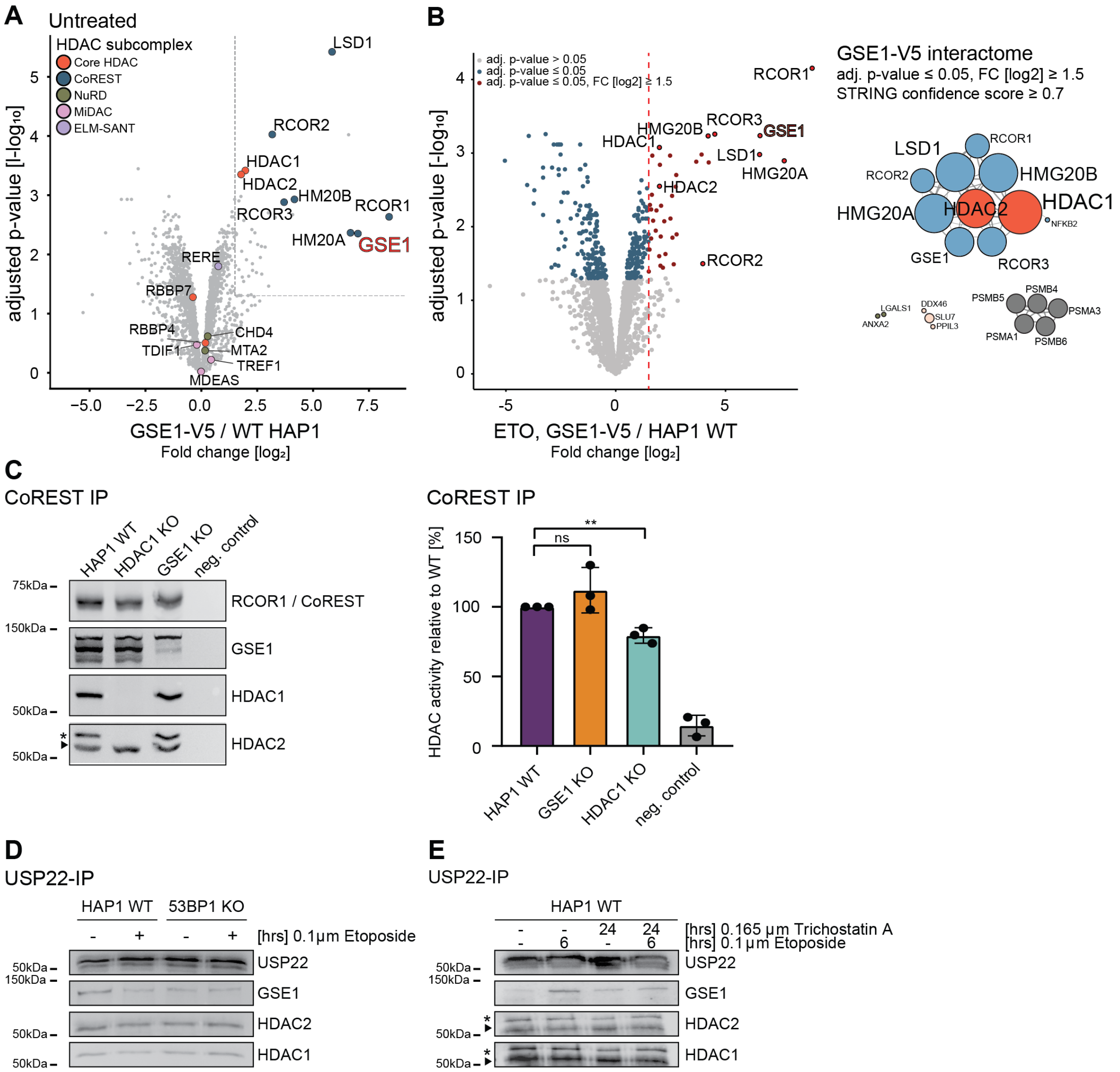

Supplementary Figure S6

(A) Volcano plot displaying the interactome of GSE1-V5 in HAP1 cells determined by AP-MS. Gene names of known subunits of the HDAC1 corepressor complexes are highlighted, such as CoREST, SIN3-related or NuRD. GSE1 is highlighted with bold red text. **(B)** Left: Volcano plot displaying the interactome of GSE1-V5 in HAP1 cells treated with etoposide (0.1 µM, 6 hours) determined by AP-MS. Significant interactors (≥ 1.5-fold change [FC] over wildtype cells [HAP1 WT], adjusted p-value ≤ 0.05) in GSE1 V5-tagged cells are shown with red dots. Gene names of known subunits of the HDAC1/CoREST complex are highlighted, GSE1 is highlighted with bold red text. Right; protein interaction network of the GSE1 interactome in etoposide-treated cells (0.1 µM, 6 hours), based on STRING DB with a confidence cut-off score of ≥ 0.7. Disconnected nodes are not displayed. Nodes are coloured according to modularity classes, and the strength of the associations is represented by the thickness of the edges. **(C)** Western blot illustrating immunoprecipitated endogenous CoREST in HAP1 WT, GSE1 KO or HDAC1 KO cells. Antibodies used for detection are depicted on the left. Asterisk: unspecific band. Negative control: pull-downs performed using anti-rabbit IgA antibodies. Impact of GSE1 deletion on the deacetylase activity of GSE1-CoREST complexes is shown in the bar graph. The data represent the mean values ± standard deviation (SD) of 3 biological replicates. Deacetylation activity obtained with HAP1 WT extracts was set to 100%. Significance is determined by unpaired parametric t-test by comparison to wildtype. *$p < 0.05$, **$p < 0.01$, ***$p < 0.001$. **(D)** Western blot illustrating immunoprecipitated endogenous USP22 from isolated HAP1 WT and 53BP1 KO cells. Antibodies used for detection are indicated on the right. **(E)** Western blot depicting immunoprecipitated endogenous USP22 purified from HAP1 WT cells treated with trichostatin A and/or etoposide for the indicated durations. Antibodies used for detection are listed on the right.

Supplementary Table legends

Supplementary Table S1. Interactome of FLAG-tagged HDAC1 in HAP1 cells

List of proteins that have been co-purified in anti-FLAG AP/MS experiments. HDAC1-FLAG was purified from HAP1 cells (HDAC1-Flag). CTRL: HDAC1 KO cells. The table depicts MS/MS counts, log2 raw intensities and log2 normalised LFQ intensities. Missing LFQ values were imputed. The table also includes results of a differential expression (DE) analysis providing e.g., fold change (FC) and adjusted p-values for proteins enriched in the HDAC1-FLAG samples.

Supplementary Table S2. Interactome of FLAG-tagged HDAC1 in MEF cells

Summary of proteins that have been co-purified in anti-FLAG AP/MS experiments. HDAC1-FLAG was purified from MEFs (HDAC1-Flag). CTRL: untagged cells. The table depicts MS/MS counts, log2 raw intensities and log2 normalised LFQ intensities. Missing LFQ values were imputed. The table also includes results of a differential expression (DE) analysis providing e.g., fold change (FC) and adjusted p-values for proteins enriched in the HDAC1-FLAG samples.

Supplementary Table S3. Acetylome data

Summary of quantified acetylation sites over MS shotgun experiments of our study. The table includes log2 ratios of site to protein normalised TMT reporter ion intensities of quantified acetylation sites as well as results of the DE analysis. Conditions measured: untreated and treated with 0.1 µM etoposide for 6 hours. Backgrounds used: HAP1 WT, GSE1 KO, HDAC1 CI. Results from Beli *et al.*, 2012 (48) are shown in column “Ratio H/L Etoposide [log2]”.

Supplementary Table S4. Phosphorylome data

Summary of quantified phosphorylation sites over MS shotgun experiments of our study. The table includes log2 ratios of site to protein normalised TMT reporter ion intensities of quantified phosphorylation sites as well as results of the DE analysis. Conditions measured: untreated and treated with 0.1 µM etoposide for 6 hours. Backgrounds used: HAP1 WT, GSE1 KO, HDAC1 CI. Results from Beli *et al.*, 2012 (48) are shown in column “Ratio H/L Etoposide [log2]”.

Supplementary Table S5. Interactome of V5-tagged GSE1 in HAP1 cells

Summary of proteins that have been co-purified in anti-V5 AP/MS experiments. GSE1-V5 was purified from HAP1 cells. CTRL: untagged HAP1 WT cells. Tested conditions: untreated and treated with 0.1µM etoposide for 6 hours.
